# Clinical utility of various liquid biopsy samples for the early detection of ovarian cancer: a comprehensive review

**DOI:** 10.3389/fonc.2025.1594100

**Published:** 2025-07-01

**Authors:** Yueqi Feng, Wanjun Yang, Jingyu Zhu, Shirui Wang, Ningjuan Wu, Hanzhi Zhao, Xiaofeng Yang

**Affiliations:** ^1^ Department of Gynecology and Obstetrics, First Affiliated Hospital of Xi’an Jiaotong University, Xi’an, Shaanxi, China; ^2^ School of Medicine, Northwest University, Xi’an, Shaanxi, China

**Keywords:** ovarian cancer, early detection, liquid biopsy, biomarkers, multi-omics

## Abstract

Ovarian cancer (OC) is a highly lethal gynecologic malignancy because of the absence of specific early symptoms and reliable biomarkers, most OC patients are often diagnosed at advanced stages, resulting in poor prognosis. Traditional tissue biopsy and serological biomarkers like CA125 have limited clinical application. Therefore, there is an urgent demand for effective diagnostic and screening tools in clinical practice. Liquid biopsy is a non-invasive method for early cancer detection by analyzing tumor-associated components shed into different body fluids, for example, circulating tumor DNA (ctDNA), circulating tumor cells (CTCs), cell-free RNA, proteins, and metabolites. Increasing evidence has demonstrated that liquid biopsy is promising for detecting cancer at an early stage. In this review, we outline the results for the utility of each liquid biopsy fluid, including serum/plasma, urine, cervical/vaginal sample, uterine lavage, and summarize the advantages and current constraints associated with their application in clinical settings. Future directions and challenges are also highlighted, along with areas where more research is warranted.

## 1 Introduction

Global Cancer Statistics reported that ovarian cancer (OC) was the 8th most frequently occurring cancer and the leading cause of cancer-related death in 2022, approximately 324,398 new ovarian cancer cases and 206,839 deaths occurred (1). The prognosis for ovarian cancer is poor because most OC (58%) are diagnosed at an advanced stage (III or IV;) (2).

OC is an extremely heterogeneous cancer. Epithelial ovarian cancer (EOC) is the most predominate pathological type, accounting for 90% of cases, which are classified into high-grade serous (up to 75%), low-grade serous (<5%), endometrioid (~10%), clear cell (~6%), mucinous (<5%) (3). A binary classification system divides invasive cancer into two types. Type I tumors are low-grade, some (endometrioid, mucinous, and clear cell types) harbor mutations in BRAF, KRAS, and PTEN with microsatellite instability, which are more indolent, less invasive. These tumors can be diagnosed at earlier stages of the disease (stage I-II). In contrast, Type II tumors included high-grade serous ovarian cancer (HGSOC), carcinosarcoma, and undifferentiated carcinomas, which are aggressive, highly genetic instability, and are mostly diagnosed at advanced stages (stage III-IV;). They are associated with high TP53 mutations, somatic and germline BRCA1/2 mutations, and other homozygous recombination genes mutations (4, 5).

Ovarian cancer has a high mortality rate primarily due to its asymptomatic nature during early stages. Most patients are diagnosed at an advanced stage when the tumor has already spread extensively. Thus, the survival rate of ovarian cancer is highly correlated with the stage at primary diagnosis. According to studies, the 5-year survival rate for early-stage disease is 92%, whereas for late-stage disease it is only 29% (6). The absence of effective screening methods and reliable biomarkers hampers early detection. Cancer antigen 125 (CA125) is considered the most useful diagnostic serum biomarker for ovarian cancer and is often used in combination with transvaginal ultrasound (TVUS) as a screening tool for detecting the disease. Currently, general population screening for ovarian cancer is not recommended because the UK Collaborative Trial of Ovarian Cancer Screening (UKCTOCS), the largest multicenter, randomized, controlled ovarian cancer screening trial to date, did not have a mortality benefit (2). However, this trial provides the first evidence that screening can detect high-grade serous tubo-ovarian cancer earlier than no screening and improve short-term treatment outcomes. The potential survival benefit was small, most likely attributed to modest gains in early detection and treatment improvements (7). This indicates that novel techniques that can detect more women with high-grade serous cancers earlier, combined with treatment improvements and a better understanding of tumor biology, may achieve a mortality benefit.

Histopathologic examination is the gold standard for OC diagnosis. However, the tumor size is usually small in early-stage patients, making puncture difficult. Additionally, transabdominal tissue biopsy is highly invasive and may lead to intra-abdominal dissemination of tumor cells. Liquid biopsy has developed rapidly over the past decades. It involves using certain biological fluids as samples to analyze and identify tumor-specific components through various omics-based detection methods. The most important advantages of liquid biopsy over traditional tissue biopsy are less invasive and can be repeated during follow-up, providing a systematic and complete response to the disease by obtaining consecutive samples for dynamic monitoring. This review systematically evaluates the evolution of biomarkers for early OC diagnosis based on different sample types used in liquid biopsy, provides a comprehensive comparison of their respective advantages and limitations across multiple dimensions, and offers theoretical foundations for optimizing early OC screening and detection strategies.

## 2 Liquid biopsy in the early detection of ovarian cancer

Liquid biopsy is gaining attention as a less invasive and more efficient alternative to traditional tissue biopsy for cancer monitoring due to its real-time results and shorter reporting time, which helps in cancer diagnosis, prognosis, monitoring disease progression, selecting appropriate treatment, and identifying drug resistance (4, 8).

**Figure 1:**
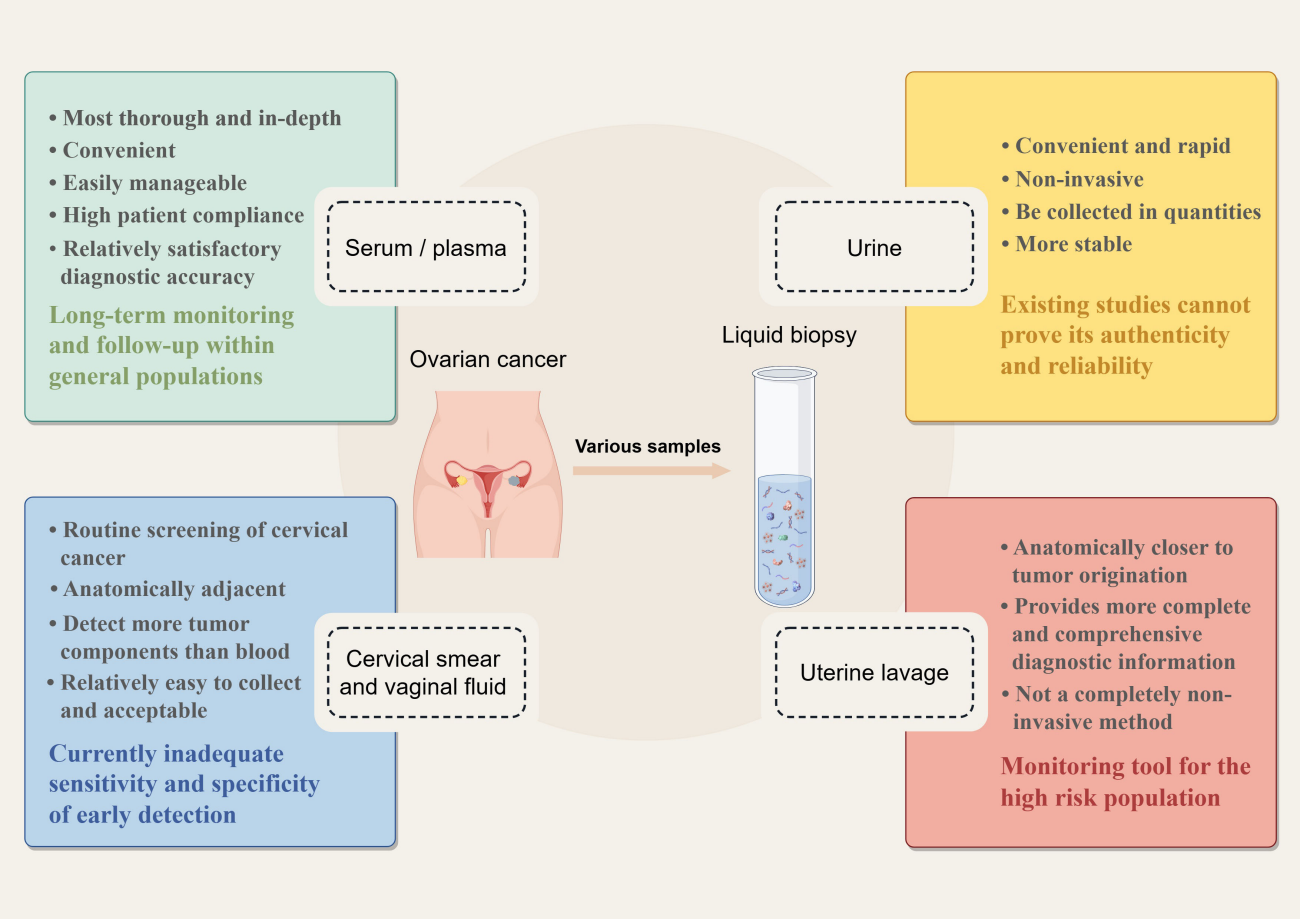
Liquid biopsy samples in the early detection of ovarian cancer (By Figdraw). The most important merits of liquid biopsy over traditional tissue biopsy are that it is minimally invasive and can be repeated several times during follow-up. For ovarian cancer, many kinds of samples have been used to detect ovarian cancer early, including serum/ plasma, urine, cervical smears, cervicovaginal mucus, and novel uterine lavage fluid. Each sample has its unique characteristics.

Various types of samples used for liquid biopsies are related to early diagnosis of OC, such as conventional serum and plasma, urine, Pap smears, cervicovaginal mucus, and uterine lavage fluid. Each of the different liquid biopsy specimens has its characteristics. **Figure 1** provides a comprehensive list of samples utilized in the current research on ovarian cancer diagnostics, highlighting their various advantages and disadvantages. In these different body fluid samples, researchers have identified many tumor-associated components using multiple omics technologies, that is, genomics, transcriptomics, proteomics, and metabolomics. These tumor-associated constituents can be used as biomarkers for the early detection of OC, including circulating tumor DNA (ctDNA), circulating tumor cells (CTCs), cell-free RNA (cfRNA), tumor-specific proteins, and metabolites. Recently, several novel components have been identified for early diagnosis through liquid biopsy, such as tumor-educated platelets (TEPs) and nano-biosensor-detected immune signals from tumor-associated neutrophils (TANs) (9). Starting from different fluid samples, we describe the collection methods of these samples and summarize the research progress of different kinds of biomarkers in different fluid samples. Then, we compare the diagnostic performance of different biomarkers from the perspective of detection technology and sample source. The aim is to identify the most appropriate method to be used for the early management of ovarian cancer.

### 2.1 Conventional serum/plasma

Blood samples have been widely and intensively studied as a conventional source of liquid biopsies over the past decades. With its convenience and accessibility, it has become more acceptable to patients. Approximately 2–10 ml of peripheral blood is collected from cancer patients, after which the plasma or serum is isolated for further study. With advances in molecular biology testing, technological innovations have emerged in genomic, transcriptomic, proteomic, and metabolomic assays, leading to the discovery of numerous novel biomarkers. **Table 1** presents the diverse biomarkers and their diagnostic efficacy in various studies using serum/plasma-based liquid biopsies.

**Table 1:** Early detection of ovarian cancer with conventional plasma/serum sample.

Analyte	Author, Year	No. of patients	Laboratory Technique	Biomarker /signature	Detection Rate
cfDNA / ctDNA	Li G et al., 2024 (10)	754 EOC + 1118 HCs	BERT, NGS, ddPCR	MethylBERT-EOC diagnostic model, OV1 ddPCR assay	MethylBERT-EOC diagnostic model: Training set: Se=93.24%, Sp=95.3%, AUC=0.98 Validation set: Se=89.24%, Sp=94.39%, AUC=0.97 OV1 ddPCR assay: Training set: Se=77.4%, Sp=92.59%, AUC=0.912 Validation set: Se=72.16%, Sp=92.95%, AUC=0.877
	Gaillard DHK et al., 2024 (11)	50 OC + 50 BOT	sWGS	bCPA score + HE4	Se=99%, Sp=42%, AUC=0.80
	Chen L et al., 2023 (12)	27 malignant OC +17 nonmalignant OC	LC-WGS	CNV-based risk predictive model ( DrCfCNV)	Se=88.89%, Sp=88.24%, AUC=0.91
	Zhou H et al., 2023 (13)	59 OC + 100 HCs	Low-pass WGS	OCscore: CNV, 5’-end motifs, fragmentation profiles, and NF	Se=94.74%, Sp=98.00%, AUC=0.98
	Buckley DN et al., 2023 (14)	128 HGSOC + 100 benign ovarian masses	RRBS, hybridization probe capture, ML	OvaPrint™: cfDNA methylation test.	Se=84.20%, Sp=96.00%, AUC=0.94
	Marinelli LM et al., 2022 (15)	91 OC + 91 healthy control	RRBS, qMSP, TELQAS	11-MDM panel	Se=96.00%, Sp=79.00%, AUC=0.91
	Liang L et al., 2022 (16)	104 OC + 56 HCs + 56 BOD	ELSA-seq	OC-D model: 18 DMRs	Training set: Se=95.70%, Sp=94.00%, AUC=0.99 Validation set: Se=94.70%, Sp=88.70%, AUC=0.90
	Bahado-Singh RO et al., 2022 (17)	5 OC + 12 controls	Genome-wide epigenetic analysis	CpG markers	4-marker predictive model: Se=94.00%, Sp=98.60%, AUC=0.97 6 AI modles: Se=100.00%, Sp=72.00-98.00%, AUC=0.99
	Faaborg L et al., 2021 (18)	79 OC	sense-antisense ddPCR	Methylated HOXA9 ctDNA	59.5% (47/79)
	Singh A et al., 2020 (19)	70 matched OC + HC	Multiplex MethyLight assay	methylation status of HOXA9 and HIC1	Se=88.90%, Sp=100.00%, AUC=0.95
	Ogasawara A et al., 2020 (20)	306 OC	ddPCR	somatic PIK3CA or KRAS mutations	27% (85/306)
	Miller BF et al., 2020 (21)	26 OC + 41 HCs	EpiClass	ZNF154 methylation density	Se=83.00%, Sp=63.00%, AUC=0.67
	Li S et al., 2020 (22)	17 OC + 15 BOT+15 HCs	MSP	Methylation of hTERT	Co=70.59%, Se=76.9%, Sp=50%
	S SK et al., 2019 (23)	72 EOC + 15HCs	MSP	Methylation of RASSF1a and BRCA1	N/A
	Cristiano S et al., 2019 (24)	28 OC	LC-WGS	DELFI: tumor-specific abnormalities in cfDNA fragmentation	Se=89%, Sp=98%
	Cohen JD et al., 2018 (25)	54 OC	multiplex-PCR	CancerSEEK: protein biomarkers + genetic biomarkers	Se=98%, Sp>99%
	Widschwendter M et al., 2017 (26)	41 OC + 37 other cancer + 5 non-epithelial tumors + 27 borderline + 19 BOT + 21 HCs	ultra-deep BS sequencing	DNAme assay: the combination of 3 regions	Se=41.4%, Sp=90.7%
	Wang B et al., 2017 (27)	71 EOC + 43 BOT + 80 HCs	MSP	Methylation of OPCML	Se=90.14%, Sp=91.87%
	Vanderstichele A et al., 2017 (28)	68 AM + 44 HCs	LC-WGS	Genome-wide z-scores: chromosomal instability	Se=74%, Sp=91%
	Phallen J et al., 2017 (29)	42 EOC	TEC-seq, NGS	somatic mutations	71%
	Parkinson CA et al., 2016 (30)	40 HGSOC	digital PCR	TP53MAF	86% (6/7)
	Cohen PA et al., 2016 (31)	32 HGSOC + 32 BOD	DNA sequencing and whole genome NIPT	CNVs	Se=40.6%, Sp=93.8%
	Wang B et al., 2015 (32)	114 EOC	nested MSP	Methylation of RUNX3, TFPI2 and OPCML	Se=90.14%, Sp=91.06%
	Shao X et al., 2015 (33)	36 OC + 22 BOD + 19 HCs	bDNA	cfDNA level	Se=88.9%, Sp=89.5%, AUC=0.917
	Pereira E et al., 2015 (34)	22 OC	WES, ddPCR	ctDNA level	Se=81%, Sp=99%, AUC=0.80
	Wu Y et al., 2014 (35)	47 EOC + 14 BOT + 10 HCs	MSP	RASSF2A hypermethylation	36.2% (17/47)
	Zhang Q et al., 2013 (36)	87 EOC + 53 BOT + 63 HCs	Multiplex-MSP assay	Methylation of APC, RASSF1A, CDH1, RUNX3, TFPI2, SFRP5 and OPCML	Se=90.57%, Sp=89.66%
	Forshew T et al., 2012 (37)	38 OC	Tam-Seq	TP53 mutation	53% (20/38)
CTCs	Wang T et al., 2022 (38)	160 OC + 90 HCs	immunomagnetic based, Multiplex RT-PCR	EpCAM, MUC1 and WT1	Se=79.4%, Sp=92.2%
	Ma J et al., 2021 (39)	156 EOC	CanPatrol System, 7 ML models	CTC counts and M-CTC percentage	AUC=0.96
	Zhang X et al., 2018 (40)	109 EOC	Multiplex RT-PCR	CTCs detection	90% (98/109)
	Rao Q et al., 2017 (41)	23 EOC + 16 HCs	microfluidic system with immunomagnetic based	CTCs detection	87% (20/23)
	Pearl ML et al., 2014 (42)	129 EOC + 48 HCs	Flow cytometry, microscopic characterization	iCTCs	Se=83%, Sp=95.1%
miRNA	Gahlawat AW et al., 2022 (43)	100 OC + 45 BOD + 99 HCs	qRT-PCR, NGS	7 cf-miRNAs panel (miR-92a, -200c, -320b, -320c, -335, -375, -486)	AUC=0.87 7-miR + CA125: AUC=0.97
	Kumar V et al., 2021 (44)	51 EOC + 14 HCs	MeDIP-NGS; qRT-PCR	3 miRNA panel (miR-205, -200c, -141)	stage I–II: Se=90.5%, Sp=100%, AUC=0.952
	Elias KM et al., 2017 (45)	164 OC + 15 controls	NGS, qPCR, neural network analysis	14 miRNAs neural network model	Training set: AUC=1.00 Testing set: Se=75%, Sp=100%, AUC=0.93
	Zheng H et al., 2013 (46)	360 OC + 200 HCs	qRT-PCR	miR-205 and let-7f	Se=71.3%, Sp=82%, AUC=0.813
	Kan CW et al., 2012 (47)	28 SEOC + 28 HCs	miRNA microarray, qRT-PCR	miR-200a, b, c and miR-182	miR-200b + c: Se=78.6%, Sp=46.4%, AUC=0.0.784
	Liu CN et al., 2020 (48)	185 EOC + 43 HCs	qRT-PCR	LOXL1-AS1	Se=65.3%, Sp=68.2%, AUC=0.843
	Li L et al., 2023 (49)	46 OC + 33 BOD	Small RNA-Seq, qRT-PCR	The sEVmiR-EOC RiskScore (miR-1246, -141-3p, -200a-3p, -200b-3p, -200c-3p, -203a-3p, and -429)	Se=87.5%, Sp=92.3%, AUC=0.913
	Zhu Z et al., 2022 (50)	36 OC + 31 BOT + 32 HCs	qPCR	miR-205	Se=66.7%, Sp=78.1%, AUC=0.715 miR-205 + CA125: Se=96.9%, Sp=83.3%, AUC=0.930
	Wang W et al., 2022 (51)	31 HGSOC + 24 HCs	Small RNA-seq	OC EV miRNA (OCEM): 8 EVs miRNAs panel (miR-1246, -1290, -483, -429, -34b-3p, -34c-5p, -145-5p, -449a)	Training set: AUC = 0.9762 Validation set: AUC = 0.9375
	Su YY et al., 2019 (52)	50 OC + 50 BOT + 50 HCs	qRT-PCR	miR-1307 and miR-375	AUC=0.874 + CA125: AUC=0.977
	Kim S et al., 2019 (53)	39 HGSOC + 10 BOD + 10 BOT +9 non-HGSOC	qRT-PCR	miRNA-145 and miRNA-200c	miRNA-145: Se=91.7%, Sp=75.0%, AUC = 0.910 miRNA-200c: Se=72.9%, Sp=90.0%, AUC = 0.802
	Yoshimura A et al., 2018 (54)	62 EOC + 26 BOT + 20 HCs	miRNA microarray, qRT-PCR	miR-99a-5p	Se=85%, Sp=75%, AUC=0.88
	Kobayashi M et al., 2018 (55)	70 OC + 13 HCs	miRNA microarray, qRT-PCR	miR-1290	Se=63%, Sp=85%, AUC=0.71
	Pan C et al., 2018 (56)	106 EOC + 8 BOT + 29 HCs	qRT-PCR	miR-21, miR-100, miR-200b	miR-21: Se=61%, Sp=82% miR-100: Se=62%, Sp=73% miR-200b: Se=64%, Sp=86%
	Meng X et al., 2016 (57)	163 EOC + 20 BOD + 32 HCs	qRT-PCR, ELISA	miR-200a, miR-200b, miR-200c	Se=89%, Sp=93%, AUC=0.95
Protein	Lyu W et al., 2024 (58)	294 OC + 63 BOT	DiSC	Spondin-1 (SPON1)	Early stages: Se=68.42%, Sp=87.30%, AUC=0.8187 All stages: Se=64.62%, Sp=87.30%, AUC=0.8255
	Galan A et al., 2023 (59)	41 OC + 127 BOD + 32 other cancers	ELISA	GD2+GD3+age	Se=97.6%, Sp=91.2%, AUC=0.976
	Gyllensten U et al., 2022 (60)	97 OC + 51 BOT	PEA Explore assay	3 models based on 4 to 7 proteins	AUC>0.96
	Enroth S et al., 2019 (61)	90 OC + 106 BOT + 28 borderline	PEA assay	11 proteins panel + age	Se=85%, Sp=93%, AUC=0.94
	Jo A et al., 2023 (62)	37 HGSOC + 14 HCs	SAViA assay	EV_HGSOC_ score:EpCAM, CD24, VCAN, HE4 and TNC	Se=89%, Sp=93%, AUC=0.95
	Peng P et al., 2019 (63)	10 OC + 10 BOD	iTRAQ, HPLC/MS	8 proteins panel	N/A
Metabolites	Fahrmann JF et al., 2024 (64)	284 OC + 550 HCs	UPLC/Q-TOF MS	7 metabolites + CA125	Discovery set: Se=86.2%, Sp=98.5%, AUC=0.98 Validation set: Se=73.8%, Sp=91.4%, AUC=0.91
	Ban D et al., 2024 (65)	431 OC + 133 HCs	UPLC-MS, RFE, CV	A consensus ML-based classifier	Accuracy=93%
	Irajizad E et al., 2022 (66)	219 OC + 190 BPM	UPLC/MS, DL	A 7-marker metabolite panel (7MetP)	AUC=0.85
	Yang W et al., 2018 (67)	47 OC + 48 HCs	UPLC/Q-TOF MS	2-piperidinone and 1-heptadecanoyl-glycerophospho-ethanolamine	Discovery set: Se=96.7%, Sp=66.7%, AUC=0.894 Validation set: Se=93.3%, Sp=80.0%, AUC=0.956
	Fan L et al., 2016 (68)	21 early EOC + 31 HCs	UPLC/Q-TOF MS	18 metabolites	AUC=0.920
	Cheng Y et al., 2016 (69)	21 OC + 17 BOT + 20 HCs	UHPLC–MS/MS	maltose, maltotriose, raffinose, and mannitol	Arabitol: AUC=0.911 4 metabolites panel: AUC=0.832
	Buas MF et al., 2016 (70)	50 serous OC + 50 serous benign controls	LC-Q-TOF-MS	4 lipid metabolites	AUC=0.85 4 lipid metabolites + CA125: AUC=0.91
	Gaul DA et al., 2015 (71)	46 early-stage serous EOC + 49 HCs	UPLC-MS, ML	16 metabolites	Se=100%, Sp=100%
	Ke C et al., 2015 (72)	140 EOC + 158 BOT+ 150 UF	UPLC-MS	53 metabolites	EOC VS. BOT: AUC=0.910 EOC VS. UF: AUC=0.9428
	Zhang T et al., 2012 (73)	80 EOC + 90 BOT	UPLC/Q-TOF/MS	6 metabolites	Se=62.5%, Sp=96.7%, AUC=0.86
	Fan L et al., 2012 (74)	80 OC + 93 HCs	UPLC/Q-TOF/MS	8 metabolites	Se=92.1%, Sp=88.6%, AUC=0.941
	Chen J et al., 2011 (75)	29 EOC + 28 BOT + 27 HCs	UPLC/Q-TOF/MS	CPG	Se=67%, Sp=77%, AUC=0.747
	Garcia E et al., 2011 (76)	170 EOC + 182 HCs	1H-NMR metabolomics analysis	4-variable model	Se=95%, Sp=68%, AUC=0.949
TEP RNA	Gao Y et al., 2023 (77)	761 adnexal masses + 167 HCs	platelet RNA-sequencing	TEPOC: 102 platelet RNAs	AUC=0.918
	Pastuszak K et al., 2021 (78)	28 OC + 30 BOD	platelet RNA-sequencing	imPlatelet	AUC = 0.95
Platelet protein	Lomnytska M et al., 2018 (79)	57 EOC + 57 BOD	MS/MS	9 protein panel	FIGO I-II: Se=83%, Sp=76% FIGO III-IV: Se=60%, Sp=83%

HCs, healthy controls; Se, Sensitivity; Sp, specificity; BERT, bidirectional encoder representations from transformers; BOT, borderline ovarian tumor; sWGS, shallow whole-genome sequencing; bCPA, benigh-calibrated copy number profile abnormality; LC-WGS, low-coverage whole-genome sequencing; RRBS, reduced representation bisulfite sequencing; ML, machine learning; qMSP, quantitative methylation specific PCR; TELQAS, Target Enrichment Long-probe Quantitative Amplified Signal; MDM, methylated DNA marker; BOD, benign ovarian disease; ELSA-seq, Enhanced linear-splinter amplification sequencing; DMRs, differentially methylated regions; ddPCR, droplet digital PCR; Epiclass, a methylation density binary classification; TF, tumor fraction; BOT, benign ovarian tumor; Co, consistency; BS, bisulfite sequencing; TEC-seq, targeted error correction sequencing; NGS, next-generation sequencing; TP53MAF, The TP53 mutant allele fraction; NIPT, non- invasive prenatal testing platform; bDNA, branched DNA; WES, whole exome sequencing; Tam-Seq, tagged-amplicon deep sequencing; M-CTC, mesenchymal–CTC; iCTCs, invasive subpopulation of CTCs; DiSC, digital immunoassay on a SlipChiP; PEA, proximity extension assay; iTRAQ, isobaric tags for relative and absolute quantitation; HPLC/MS, high-performance liquid chromatography/mass spectrometry; UPLC/Q-TOF MS, ultra-performance liquid chromatography and quadrupole time-of-flight mass spectrometry; RFE, recursive feature elimination; CV, cross-validation; BPM, benign pelvic masses; DL, deep learning; UF, uterine fibroid; CPG, 27-nor-5β-cholestane-3,7,12,24,25 pentol glucuronide; TEPOC, TEP-derived gene panel of ovarian cancer.

#### 2.1.1 Cell-free DNA/circulating tumor DNA

Cell-free DNA (cfDNA) is released into the bloodstream mainly through apoptosis or necrosis. Elevated levels of cfDNA are observed in OC patients compared to healthy individuals, making it a valuable tool for early cancer diagnosis and screening (33, 80). Circulating tumor DNA (ctDNA), a subset of cfDNA, carries genetic and epigenetic information specific to the tumor, providing a "real-time" snapshot of the disease burden (81). With the advancement of sequencing technologies, researchers have shifted their focus toward identifying tumor-associated genetic mutations (29, 30, 34, 37), analyzing their methylation status, and performing DNA fragment mapping analysis (24) as well as CNV-based genomic instability screening (12, 13, 28, 31).

The detection rate of TP53 mutant-ctDNA in HGSOC patients is relatively high, ranging from 75% to 100% (82). Previous studies of TP53 mutations in EOC patients have demonstrated high sensitivity (>75%) and specificity (>80%) for recognizing ctDNA mutations (30, 34, 37). However, a majority of research on tumor-associated mutations involves the prior identification of tumor-specific mutations through tumor or formalin-fixed paraffin-embedded (FFPE) tissue, followed by the development of assays for these specific mutations. This approach limits such studies to the theoretical stage, as tumor tissue-specific mutations cannot be determined before diagnosis. For this reason, Phallen et al. developed targeted error correction sequencing (TEC-Seq) to allow ultra-sensitive direct assessment of serial changes in cfDNA by massively parallel sequencing, without the need for prior knowledge of genetic alterations in the tumor. This platform increases the sensitivity to 97.4% and the specificity to 100% (29).

Increased methylation of promoter regions is thought to be an early epigenetic event during tumorigenesis that can transform the expression of tumor suppressor genes. Methylation of cytosine occurs at relatively stable modified cytosine-phosphate-guanine (CpG)-rich regions (CpG islands) of DNA (83, 84). Several studies found that DNA methylation can be a promising biomarker for OC diagnostic, therapeutic, and prognostic (85). A comparative systematic review of 29 articles identified RASSF1A, BRCA1 (23, 35), and OPCML as common gene-specific methylation biomarkers, with OPCML showing the best diagnostic performance and an optimal sensitivity of 97.8% (86). While methylation panels consisting of 2 or more genes, the combination of different regions and CpGs had better diagnostic performance, with sensitivity ranging from 84.2% to 94.7%, and specificity ranging from 86.7% to 100% (15, 16, 19, 32, 36, 86, 87). Among these studies, Zhang et al. identified seven candidate genes using multiplex methylation-specific PCR (MSP), with 85.3% sensitivity and 90.5% specificity for stage I EOC (36). OvaPrint™ is a cfDNA methylation-based liquid biopsy platform to discriminate benign pelvic masses from HGSOC preoperatively. By leveraging machine learning to analyze sequencing data, researchers constructed this classifier (OvaPrint™), which achieves a positive predictive value of 95% (14). As for the combination of AI with several different CpG markers, statistical and bioinformatics approaches yielded high diagnostic accuracy with an AUC close to or equal to 1.0 (17). Li et al. employed transformer-based AI technology to learn genome-wide methylation patterns among different CpG sites from 110,000 cancer samples. These features were then applied to analyze large-scale cfDNA methylation markers in 754 EOC patients (including 205 early-stage EOC patients) and 1,118 healthy female controls. Using a pretrained AI transformer system named MethylBERT, they developed an EOC diagnostic model, which achieved a sensitivity of 80% and a specificity of 95% in early-stage EOC detection (10). Unlike most previous studies that relied on conventional modeling strategies, where genetic or epigenetic differences in cfDNA between cancer patients and healthy controls were directly analyzed to build diagnostic models, this study employed a pretrained AI methylation transformer system. Traditional methods like LASSO regression were constrained by the events-per-variable (EPV) rule, limiting the number of input markers that could be incorporated. In contrast, this pretrained AI transformer system is unrestricted by input feature, making it an ideal choice for constructing cfDNA-based diagnostic models. Sequencing data combined with artificial intelligence algorithm analysis could be a big step towards the early detection of ovarian cancer.

Recent advancements in detection technology have produced highly sensitive methods such as digital PCR (dPCR) and droplet digital PCR (ddPCR). Next-generation sequencing (NGS) allows for the detection of multiple genomic regions in a single assay, facilitating DNA mutation profiling and tumor mutation load assessment. Notably, whole genome sequencing (WGS) has also significantly improved the diagnosis of copy number variations (CNV). HGSOC is characterized by high chromosomal instability. Using CNV profiles from cfDNA as biomarkers could enhance the detection of malignancy in patients with adnexal masses (28). The DrCf10CNV predictive model was formulated using a combination of the CNV panel and machine learning algorithms (12). It has a sensitivity of 89% for distinguishing between malignant and non-malignant ovarian tumors. Another integrated scoring system, termed the OC score, was developed to classify OC patients from healthy controls based on four genomic features: CNV, 5'-end motifs, fragmentation profiles, and nucleosome footprinting (NF). The system has a high precision (AUC 97.7%; sensitivity 94.7%; specificity 98.0%) as a new comprehensive diagnostic method and a satisfactory sensitivity (85.7%) for early-stage OC (13). Compared with single-dimensional methylation sequencing technology, this study used multi-dimensional variation indicators to make the OC score system with stable performance, wider coverage, and greater overall accuracy.

Despite advancements in detection technology, the biological characteristics of ctDNA hinder its ability to detect small tumors or pre-cancerous lesions. Firstly, the limited detection of low-frequency ctDNA alleles may be attributed to the fact that ctDNA is diluted in higher concentrations of non-tumor cfDNA and contaminated with high molecular weight DNA (88). Additionally, it is possible that cancers may not shed enough ctDNA to detect early-stage or micrometastatic disease due to low disease burden. It is important to note that false-positive ctDNA mutations may be caused by non-cancerous mutations. Therefore, it is necessary to have superior sensitivity and precision in detecting ctDNA in early-stage disease. Excitingly, Thusgaard CF et al. recently reported for the first time a highly sensitive and transparent tumor-informed ctDNA single nucleotide variant detection method (89). This approach combines various allelic and read depth filters with different cut-offs, introduces an additional panel of normals to eliminate background noise, and utilizes a new filtering method to enhance the detection of ctDNA signals in plasma. These advancements significantly enhance the reliability of ctDNA-based approaches for the early diagnosis of OC.

#### 2.1.2 Circulating tumor cells

Circulating tumor cells (CTCs) are thought to be detached from the primary tumor site, undergo the process of epithelial-mesenchymal transition (EMT), pass through the bloodstream, and colonize distant sites, leading to regional or distant metastasis (90). The molecular characterization and analysis of CTCs in different solid tumors exhibit variations (91). Most studies regarding CTCs in OC have focused on more advanced staging and have mainly investigated the relationship with prognosis, with fewer studies related to early diagnosis. For stage IA-IB patients, the positive rate of CTCs was 93%, which was significantly higher than the positive rate of CA125 in the same patients. However, the number of CTCs found in stage I was significantly lower than those in stage III and IV (40). Similarly, CTCs were not only found at a higher rate in advanced stages compared to early stages but also revealed a higher CTC count, 41.3 CTCs/ml versus 6.0 CTCs/ml (92).

Due to variations in CTC isolation methods and detection protocols across studies, including differences in sampling timepoints and cohort sizes, the reported CTC positivity rates in OC patients varied widely, ranging from 14% to 100% (90). Immunoaffinity-based biotechnology is the most common method for CTC enrichment. The CellSearch® system, approved by the FDA in 2004, utilizes EpCAM-targeted immunomagnetic beads to isolate CTCs from peripheral blood samples. However, the CellSearch® system demonstrates relatively low overall detection rates in ovarian cancer patients, ranging between 14.4% to 26% (93). The biggest limitation of this detection method is that EpCAM is a marker of epithelial cells and not a tumor-specific marker. Subsequently, a series of novel CTC detection technologies have emerged, including: invasive CTC subset marker detection techniques (42), high-throughput microfluidic systems integrating both physical and biological characteristics (41, 94), fluorescent-magnetic nanoparticles modified with folic acid and antifouling hydrogel (95), and flexible graphene-based biosensor for sensitive and rapid detection (96). These novel methods have significantly improved the detection rate of CTCs to over 70%. The Cell Adhesion Matrix (CAM)-based platform for detecting invasive CTCs (iCTCs) achieved a positive predictive value (PPV) of 77.8% in identifying early-stage epithelial ovarian cancer (EOC) patients (42). An optimized CTCs detection model incorporating three markers (EpCAM, MUC1, and WT1) achieved 79.4% sensitivity and 92.2% specificity (38).

Despite promising results from some studies, these findings are not yet recommended for clinical application. Some of the aforementioned novel technologies remain confined to theoretical exploration or have only been validated in small patient cohorts, limiting their generalizability to broader populations. Further clinical validation remains imperative. Research on early diagnosis is still in the initial phase, and further work is necessary for rapid, simple, and standardized assays, as well as studies targeting different subgroups with heterogeneity of CTCs. Further assessment is pivotal to determine the diagnostic performance of CTCs or specific subgroups.

#### 2.1.3 Cell-free mRNA and EVs miRNA

Rapid tumor progression leads to increased gene transcription and the release of cell-free RNAs (cfRNAs), including mRNAs and miRNAs, into circulation. MiRNAs are stable in body fluids, and their altered expression is closely associated with tumor progression, invasion, metastasis, and drug resistance (97). It was reported that miRNA expression was dysregulated in the blood of ovarian cancer patients (46, 98–101).

Cf-miRNAs are highly stable in body fluids. Early studies used qRT-PCR and miRNA microarrays for expression analysis to detect aberrantly expressed miRNAs, with AUC ranging from 0.784-0.843 (46, 47). After genome-wide analysis, more tumor-associated cf-miRNAs were identified, and detection performance could be greatly improved by constructing a miRNA panel. The three miRNA panels can achieve 90.5% sensitivity and 100% specificity for their overall diagnostic potential in early serum samples (44). The other 7 cf-miRNAs panel and the 14 miRNAs neural network model were able to achieve the AUC of 0.87 and 0.93, respectively (43, 45, 102).

Research has predominantly focused on secreted miRNAs within extracellular vesicles (EVs), primarily produced by cells, notably cancer cells. EVs, categorized as exosomes, microvesicles, and apoptotic vesicles (103), facilitate intercellular communication and influence cancer development, progression, and metastasis by transporting bioactive components like nucleic acids, proteins, metabolites, and lipids. These substances are found in circulating blood and various biofluids, and have emerged as promising non-invasive biomarkers (104). EVs carry abundant miRNAs, which are well-protected from RNase degradation. Consequently, EV-miRNAs, such as the miR-200 family (47, 57), miR-21, miR-100 (56), miR-99a-5p (54), and miR-1290 (55), are more frequently studied as potential biomarkers compared to non-exosome circulating miRNAs, for early detection of OC (105). EVs' miRNAs can facilitate paracrine and endocrine communication between different tissues, regulating gene expression and remotely modulating cellular functions (106). The sEVmiR-EOC score constructed from seven EVs miRNAs had superior diagnostic performance, especially in distinguishing patients with stage I EOC from benign ovarian tumors, with an AUC of 0.903 (specificity, 100%; sensitivity, 80%) (49). The OCEM signature, composed of eight EV mRNAs, achieved an AUC of 0.976 (51). Candidate exosomal miRNAs as biomarkers were mostly selected based on miRNA profiles (53, 56, 57) of tumors or exosomes secreted by ovarian cancer cell lines (54, 55), without a large-scale screening of patient plasma exosomal miRNAs. Therefore, the expression of miRNAs in tumor tissues might be inconsistent with the expression of circulating exosomal miRNAs. For example, miR-145 has been reported to be significantly down-regulated in EOC tissues, especially in HGSOC (107, 108). However, it was significantly up-regulated in serum exosomes from EOC patients and showed promising accuracy for EOC detection (sensitivity of 91.7%, specificity of 75%, AUC of 0.910) (53). The researchers employed a “banishing theory” to state that because miR-145 overexpression in ovarian cancer cells inhibits cancer progression, it is released from the cancer cells as exosomes. This implies that there may be undiscovered selection and sorting mechanisms that control the preferential encapsulation of specific miRNAs into exosomes before releasing them into the tumor microenvironment for intercellular communication (105).

For both cf-miRNA and EVs miRNA, the diagnostic accuracy of single miRNA is limited, but combining these miRNAs with traditional serological markers can increase the sensitivity of the assay (43). Combining exosomal miR-205 with CA125 and HE4 raised the AUC to 0.951, with sensitivity and specificity of 100% and 86.1%, respectively (50). Additionally, pairing CA125 with miR-99a-5p resulted in an AUC of 0.91 for differentiating early EOC from healthy controls (54). By combining next-generation sequencing of serum miRNAs with machine learning techniques, neural network analysis has the ability to identify more stage I/II ovarian cancers with a significantly lower false-positive rate, as well as identifying borderline tumors much better than CA125 (45). EVs' miRNA/cf-miRNA profiles may be influenced by a variety of factors. These comprise individual genetic variation, specimen source, various pre-analytical factors (including the degree of hemolysis), miRNA isolation methods, different assay platforms (e.g., RT-qPCR or NGS), different qPCR techniques (e.g., Taqman and LNA assays), and selection of standard reference genes. Similar to ctDNA, exosomes pose similar hurdles. Tumor-derived vesicles often account for less than 2% of circulating exosomes and undergo rapid clearance, necessitating high-throughput, high-sensitivity analytical methods for accurate detection. To successfully apply EVs or cf-miRNAs as biomarkers for the early diagnosis of OC in the clinical setting, these factors need to be carefully considered and standardized.

#### 2.1.4 Tumor-educated platelets

Platelets display reactive responses during tumor progression and treatment. Tumor cells can directly or indirectly alter platelet RNA and protein content, resulting in the transfer of tumor-associated biomolecules to platelets. These tumor-educated platelets (TEPs) can promote cancer cell survival and metastasis and are considered potential diagnostic tools for cancer (109).

TEPs may also undergo queue-specific splicing events in response to signals released by cancer cells and the tumor microenvironment. The combination of specific splice events in response to external signals and the ability of platelets to directly uptake circulating mRNA could provide TEPs with a highly dynamic mRNA library, with potential applicability to cancer diagnosis (109). RNA sequencing of TEPs has become the latest component of liquid biopsy for cancer detection. Through mRNA sequencing of TEPs from 283 platelet samples, Best et al. achieved 96% accuracy in distinguishing 228 patients with localized or metastatic tumors from 55 healthy controls, along with 71% accuracy in identifying primary tumor locations (110). Although this study included samples from six different types of cancer, the results proved remarkably robust and suggest potential applications for similar technology in ovarian cancer patients. Subsequently, Piek et al. demonstrated the advantages of TEPs in both diagnosing and differentiating early-stage ovarian cancer from benign tumors, achieving an accuracy of 80% (111). Researchers developed an enhanced bioinformatics approach using deep learning, termed imPlatelet. The imPlatelet classifier converts platelet RNA sequencing data into images, where each pixel corresponds to the expression level of a certain gene. This method achieved 95% accuracy in distinguishing non-cancer patients from those with ovarian cancer (78). In addition to mRNA, proteins in platelets can also serve as potential biomarkers. An extensive proteomic approach identified a 9-protein panel in TEPs, yielding an AUC of 0.831 for early OC diagnosis (79).

While the aforementioned studies were conducted in limited patient cohorts, an intercontinental, hospital-based, diagnostic study enrolled 761 treatment-naïve inpatients with histologically confirmed adnexal masses and 167 healthy controls from nine medical centers (77). The TEPOC classifier, comprising 102 platelet RNAs, demonstrated robust diagnostic performance across diverse populations and OC subtypes, achieving an AUC of 0.858 for early-stage OC detection. This study confirms the potential of OC early detection by platelet RNA. In the future, TEPs analysis with complementary ctDNA/CTC analysis and platelet quantification may become a blood-based cancer diagnostic method.

#### 2.1.5 Protein

Proteins are integral to numerous biological processes, and their post-translational modifications (PTMs), such as phosphorylation, acetylation, and glycosylation, play critical roles in cancer development and progression (112). The tumor biomarker CA125, which is routinely used in clinical practice, is a highly glycosylated mucin. Although it is the most sensitive and accurate serum biomarker in practical implementation, CA125 is still insufficient for early detection of ovarian cancer. Moreover, some benign diseases can also cause elevated CA125, such as endometriosis and pelvic inflammatory diseases. Many new biomarkers have been identified in the ongoing progress of MS-based proteomics, facilitating the development of OC multivariate index assays such as OVA1, Risk of Ovarian Malignancy Algorithm (ROMA), and Overa (113–116). These tests have greatly improved the sensitivity of OC diagnosis compared to single CA125, but have also reduced specificity to some extent. Proteomic analysis of exosomes isolated from the peripheral blood of patients with early-stage EOC and non-cancer controls identified eight proteins that could serve as potential ovarian cancer markers (63). The study initially identified upregulation of 35 proteins in both serum exosomes and tumor tissues from OC patients. Among these 35 proteins, eight of these proteins were confirmed in both exosome databases and other studies. However, validation in clinical cohorts is missing. Jo et al. developed a high-throughput EV screening platform (SAViA) to construct an EV_HGSOC_ score model containing five proteins (EpCAM, CD24, VCAN, HE4, and TNC). This model demonstrated a sensitivity of 89%, a specificity of 93%, and an AUC of 0.95. The panel was able to differentiate early-stage HGSOC from the advanced-stage and non-cancer groups with a specificity of 91% and a sensitivity of 76% (62). The Proximity Extension Assay (PEA) Explore is an ultra-sensitive proteomics technology capable of characterizing much more of the plasma proteome with very little input material. PEA technology greatly improves the detection rate by integrating the high specificity of antibody immunoassay with the high sensitivity and throughput of genomics. Combining PEA analysis and machine learning identified multi-protein panels with AUCs exceeding 0.96 (60, 61). Most proteins in these models were associated with OC. All the protein panels mentioned above include WFDC2 (HE4), which is a clinically used biomarker for ovarian cancer.

Despite methodological differences, Jo et al. established murine fallopian tube (mFT) cells with oncogenic mutations and performed proteomic analysis on mFT-derived EVs (62). In contrast, Gyllensten et al. employed PEA Explore to compare plasma proteins between benign and malignant tumor patients (60, 61). All three studies used high-throughput analytical methods and obtained robust results, suggesting that novel plasma-based biomarker candidates for ovarian cancer screening can be discovered by harnessing the power of high-precision proteomics. The integration of high-throughput sample preparation technologies and automated systems, advanced MS-based glycoproteomic research methods, AI-driven data analysis techniques, and the establishment of more comprehensive and complete databases can accelerate the discovery and application of protein-based OC biomarkers (117).

#### 2.1.6 Metabolites

Metabolites constitute the endpoints of many biofunctional molecular processes, and disturbances at the level of metabolism in the blood and/or other body fluids have long been recognized as promising indicators of cancer. Metabolic profiles have been proposed as molecular phenotypes of biological systems, reflecting pooled information encoded at the genomic level as well as responses at the transcriptomic and proteomic levels (65). Exploring the metabolic profile of OC can both assist in early diagnosis and investigate the underlying biological mechanisms of ovarian cancer (68, 118). Abnormal lipid metabolism (70, 71), fatty acid β-oxidation (67, 72), and amino acid catabolism (72, 118) are among the metabolic pathways associated with ovarian cancer progression. Metabolomics studies of ovarian cancer have mainly used nuclear magnetic resonance (NMR) (76, 119) and mass spectrometry (MS)-based methods (67, 68, 73–75). Garcia et al. applied NMR spectroscopy-based metabolomics to discriminate ovarian cancer patients at early stages from healthy controls, with an AUC of 0.949 (76). Chen et al. discovered 27-nor-5β-cholestane-3,7,12,24,25 pentol glucuronide (CPG) as a complementary diagnostic marker to CA125 with an AUC of 0.747 (75). Zhang et al. also used UPLC/Q-TOF/MS for the test, and the six metabolites model could reach an AUC of 0.86 (73), and the other eight metabolites model had an AUC of 0.941 (74). Ke et al. conducted a large-scale metabolic study of 448 plasma samples with a UPLC/MS platform. The study results identified metabolic profiles and potential biomarkers, distinguishing between EOC or early-stage EOC and benign ovarian tumors (BOT), with AUC values of 0.9100 and 0.8385, respectively (72). Combining metabolomics profiling with machine learning enables more accurate analysis of large datasets, facilitating the understanding of disease-specific variants and further biomarker discovery. A linear support vector machine (SVM) model consisting of 16 diagnostic metabolites was able to identify early OC in a patient cohort with 100% accuracy (71). Using recursive feature elimination (RFE) coupled with repeated cross-validation (CV) based on UPLC-MS metabolomics, the developed model was able to distinguish OC cases from controls with 93% accuracy. Importantly, the overall predictive accuracy of the consensus classifier was better for early-stage patients compared to advanced-stage patients (65).

#### 2.1.7 Advantages and challenges

Plasma and serum, the traditional liquid biopsy samples, have the most abundant relevant studies, demonstrating relatively satisfactory diagnostic performance. A series of sophisticated high-throughput techniques has also been prioritized in plasma/serum, enabling further enhancement of the precision of the assay. To sum up, serum/plasma testing is a convenient and readily acceptable method for the dynamic monitoring of a patient's disease progression.

However, tumor-associated components are shed from the primary tumor site, enter the bloodstream, and circulate systemically. During this process, their detection rate may be affected by the significant reduction of their number, together with the presence of non-tumor-associated components in the blood. Therefore, high-performing single biomarker and/or biomarker panels in blood samples currently rely on advanced and high-throughput technologies, most of which are expensive and do not meet the requirements of healthy economics. There is still a need for further research into potential biomarkers which would be practical and could be used for census purposes. Ahn et al. proposed that combining plasma proteomics and metabolomics can identify emerging features that are difficult to detect using a single omics approach (120). This indicates that in the future, the combination of multi-omics methods and machine learning will better facilitate the early detection of OC.

### 2.2 Urine

Urine can be used as a convenient, rapid, and completely non-invasive method of liquid biopsy for nearly all patients. Since it is a product of normal metabolism and secretion, it can be collected in large quantities. Urine has been shown to contain a variety of proteins/peptides, with approximately 70% of urinary protein derived from the kidney and 30% from plasma (121), making it a potential biomarker. Compared to blood, urine is more stable, and protein-related degradation pathways are completed by the time of excretion. In contrast, many protein hydrolysis degradation products are produced by the activation of proteases in blood samples (especially the coagulation cascade), which may interfere with the results (122). **Table 2** presents studies related to the early detection of ovarian cancer with urine samples, and we can observe that most analyses focused on proteomics and metabolomics. With regard to genomics, there are fewer reports about urine. Only one team found that transrenal DNA (TrDNA) was more indicative of DNA methylation status than serum/plasma. For the methylation of HIST1H2BB and MAGI2, the correlation between tumor DNA and TrDNA methylation measurements was stronger (123).

**Table 2:** Early detection of ovarian cancer with urine sample.

Analyte	Author, Year	No. of patients	Laboratory Technique	Biomarker /signature	Detection Rate
TrDNA	Valle BL et al., 2020 (123)	2 OC	NGS, BS, qMSP	HIST1H2BB and MAGI2	N/A
miRNA	Zhou J et al., 2015 (124)	39 EOC + 26 BGD + 30 HCs	miRNA microarray, RT-qPCR	miR-30a-5p	Upregulated: miR-30a-5p Downregulated: 37 different miRNAs
	Záveský L et al., 2015 (125)	6 EOC	RT-qPCR	miR-92a, miR-106b	Upregulated: miR-92a Downregulated: miR-106b
	Berner K et al., 2022 (126)	13 EOC + 17 HCs	RT-qPCR	miR-15a, let-7a	Upregulated: miR-15a Downregulated: let-7a
Intrinsic fluorophores	Martinicky D et al., 2015 (127)	36 OC + 35 BOT + 42 HCs	CMSS analysis	fluorescence intensity	OC vs. BOT: Se=86.11%, Sp=77.14% OC vs. HC: Se=91.67%, Sp=100%
Proteins and peptides	Woolery KT et al., 2014 (128)	32 OC + 23 BOD + 10 HCs	ELISA	IL-1β	N/A
	Smith CR et al., 2014 (129)	6 OC + 6 HCs	Nano LC-MS/MS	LRG1	N/A
	Stockley J et al., 2020 (130)	26 OC + 58 BOD	ELISA	MCM5	Se=61.5%, Sp=75.9%, AUC=0.68
	Murgan SS et al., 2020 (131)	112 OC + 200 HCs	PAGE-SDS electrophoresis and Edman degradation technique	Urinary micro-peptides	62.5% (70/112)
Metabolites	Niemi RJ et al., 2017 (132)	71 adnexal mass +22 BGD	LC-MS/MS	DiAcSpm	Se=86.5%, Sp=65.2%
	Liu X et al., 2020 (133)	150 OC + 20 BOT +81 BO	UHPLC-QTOF-MS	2 metabolites	Se=97.66%, Sp=87.50%, AUC=0.984

BGD, Benign gynecological disease; CMSS, Concentration matrices of synchronous spectra; LRG1, Leucine-rich alpha-2-glycoprotein; PAGE-SDS Electrophoresis, Polyacrylamide Gel-SDS gel Electrophoresis; DiAcSpm, Urinary N1,N12-diacetylspermine.

Urinary miRNAs are usually isolated via total RNA from extracellular vesicles and cellular components, and then small RNA molecules (<200 nt) are amplified for qRT-PCR analysis (134). The current study only identified up- or down-regulated miRNAs in the urine of OC patients and did not conduct further trials on whether these miRNAs could detect OC earlier. Zhou et al. provided the first evidence of elevated miR-30a-5p in the urine of OC patients and confirmed that the up-regulation of miR-30a-5p may be closely related to the early stage and lymphatic metastasis (124). Záveský et al. analyzed and compared preoperative and postoperative specimens from the same EOC patients, finding that urinary miR-92a was upregulated while miR-106b was downregulated (125). However, subsequent studies by this team showed that miRNAs from extracellular urine sources did not show significant differences compared to tissue and ascites samples (135). The lower levels of detectable miRNAs in urine compared to blood may account for this. It is postulated that most circulating miRNAs are reabsorbed by the kidneys through an as yet unknown mechanism and destroyed by high levels of RNase in the urinary tract (134).

Since the first analysis of serum peptidomics in OC (136), several studies have found that low molecular weight proteins and peptides in different body fluids can provide specific diagnostic information for different types of cancer (137, 138). The first study of OC-associated urinary peptidomes was performed by Smith et al. They identified several endogenous urinary peptides that could serve as potential biomarkers, the most promising of which was leucine-rich alpha-2-glycoprotein (LRG1) (129). A prospective, longitudinal, case-control study assayed and sequenced urinary micropeptides from OC patients and their age-matched volunteers, revealing that urinary catalase (CAT), alpha-1 acid glycoprotein (AAG), and peroxidase-2 (Prx-2) could serve as biomarkers for early detection of ovarian cancer and response to treatment (131). Studies have also found protein expression in the urine of OC patients that correlates with cancer cell proliferation and invasion. For instance, IL-1β and MCM5 have been identified (128, 130). *In vitro* trials have also shown differences in urinary protein expression between OC tumorigenic and non-tumorigenic rats, which have been hypothesized to be related to ovarian cancer metastasis (139). But the studies above either lacked evaluation of diagnostic tests or had insufficient diagnostic performance.

Slupsky et al. demonstrated for the first time that changes in metabolite concentrations displayed by urinary metabolic profiling may be associated with ovarian cancer specificity (140). Metabolite polyamines are present in elevated levels during the process of active cell proliferation, such as in cancer patients. Urinary N1, N12-diacetylspermine (DiAcSpm) serves as a significant diagnostic and prognostic marker in various types of cancer. A proof-of-concept study utilizing LC-MS/MS revealed elevated levels of urinary DiAcSpm in patients with malignant ovarian tumors, including those with low malignant potential and early-stage disease. DiAcSpm had a better sensitivity (86.5%) but lower specificity (65.2%) (132). One metabolomics study utilized non-targeted techniques to detect metabolite profiles in urine, and developed a model of 2 urinary putative metabolites for ovarian cancer diagnosis by the support vector machine algorithm. The AUC of the model was 0.984, with a sensitivity of 97.66% and a specificity of 87.50% (133).

Urine samples have a special feature of containing several intrinsic fluorophores that can be detected through fluorescence analysis, which is rapid, safe, and highly sensitive. Therefore, the analysis and surveillance of autofluorescence in urine present a new opportunity for ovarian cancer screening methods. Blue-fluorescing pteridines dominate in the excitation-emission matrices of cancer urine samples (141). The study found that using Concentration Matrices of Synchronous Spectra (CMSS) resulted in high sensitivity (91.67%) and specificity (100%) in differentiating between patients with ovarian malignancy and healthy women (127). There are fewer studies related to cancer with urine fluorescence. Urine autofluorescence at 295 nm was found to be significantly higher than that of healthy controls in the urine of patients with malignant melanoma at each clinical stage (142). A combination of urine fluorescence spectroscopy with machine learning algorithms has shown promising capabilities in screening for endometrial cancer (143). OC is a near-urinary tumor, along with endometrial cancer, and perhaps urine autofluorescence could also be used as a tool for OC screening.

Current studies on relevant urinary biomarkers for the detection of OC are mostly limited to a theoretical level. The authenticity and reliability of these potential biomarkers have not been evaluated. Available clinical findings are also insufficient to effectively detect early OC, though they may have greater diagnostic power when combined with other non-urinary biomarkers and imaging tests. Thus, further validation is required for urine as a liquid biopsy sample to detect OC.

### 2.3 Pap test and cervicovaginal fluid

Since the advent of the Papanicolaou (Pap) test as a routine screening tool for cervical cancer, the mortality of cervical cancer has dramatically decreased. The ovaries, fallopian tubes, uterus, and vagina are anatomically adjacent, it has been proven that tumor DNA could be detected in the vaginal tract of OC patients (144). In recent years, researchers have continued to evaluate the potential role of the Pap test in the early detection of OC. Because the cells in such samples are shed directly from the primary tumor, they harbor tumor DNA in both greater quantities and higher concentrations than the DNA in circulating in the bloodstream.

In 2013, Kinde et al. first discovered ovarian cancer mutated DNA molecules in Pap smear specimens (145). This indicated that ovarian cancer cells and cell debris were present in the cervix and could be detected by molecular genetic techniques, providing early evidence of the viability of detecting gynecologic cancers by intracervical DNA detection. This was the beginning of a chapter in the study of early diagnosis of OC using Pap test. The most commonly mutated gene in epithelial ovarian cancers was TP53 (145). TP53 has been identified as the most common gene associated with OC in purified DNA from Pap test samples. These samples were collected approximately 2.5–7 years before OC diagnosis in pre-symptomatic women (146, 147). Despite the low DNA abundance in these samples, ultrasensitive ddPCR was capable of identifying tumorigenic TP53 mutations. The mutation detection rate among tumor-associated genes, such as TP53, in Pap smear specimens from both OC patients and those with presymptomatic OC was inadequate to fulfill the screening standards. The sensitivity of detecting ovarian cancer by tumor-specific mutations in Pap test sample ranged from 33% to 75% (145–149). Only Jiang et al. showed that somatic mutations carrying tumor genomic information were tested in all Pap smears (150). The reason for this phenomenon may be related to small patient cohorts, most of which had only approximately 20 OC patients. A large cohort study with 245 OC patients and 714 healthy controls used PapSEEK. This test incorporates assays for mutations in 18 genes as well as an aneuploidy assay, has shown a sensitivity of 33%, including 34% of patients with early-stage disease (148). Deep sequencing of the TP53 gene using Duplex Sequencing (DS) on all 30 Pap test specimens only detected tumor-derived TP53 mutations in 38% of OC cancer patients (149). Combining plasma circulating tumor DNA with a Pap test has been shown to increase sensitivity (148), but it is insufficient for clinical application. Despite the use of more sophisticated techniques, higher sensitivity assays, and larger cohorts, the mutation detection rate has not substantially improved. This may be due to the anatomical distance between the collection site (cervical canal) and the fallopian tube, which is thought to be the origin of serous ovarian cancer, rather than technical limitations. The improved detection sensitivity achieved with Tao brushes further supports this hypothesis (148).

Besides detecting DNA mutations, Chang et al. demonstrated the feasibility through DNA methylation analysis of cervical scrapings. The hypermethylation of POU4F3/MAGI2 was observed in both OC tissue and cervical scrapings, with a sensitivity and specificity of 61% and 62%-69%, respectively (151). Then, the same team thoroughly investigated OC-specific DNA methylation biomarkers in conventional Pap tests, analyzed the methylomes of tissues and cervical scrapings, and integrated public methylomics datasets to depict methylomics profiles. An OC risk prediction model comprising AMPD3, NRN1, and TBX15, achieved a sensitivity of 81%, a specificity of 84%, and OC detection accuracy of 91% (152).

In the current study, the investigators chose to focus on genomic instability instead of somatic gene mutations. A pattern of genomic instability has been demonstrated in the early stages of HGSOC progression (153). Copy number aberrations (CNA), a form of chromosomal instability, are the most prevalent structural variation in the genome. Unlike single nucleotide variants, somatic copy number alterations (SCNAs) are rarely found in normal tissues, though they are common in cancer (especially in HGSOC) (154). A recent retrospective and multicenter cohort study analyzed 250 archived Pap test DNA collected before diagnosis. Researchers derived the copy number profile abnormality (CPA) from Pap test samples using low-pass whole-genome sequencing. They integrated the CPA score into the EVA (early ovarian cancer) test, achieving sensitivity of 75%, specificity of 96%, and accuracy of 81% (155). The detection of characteristic DNA methylation and genomic instability in Pap test specimens has greatly improved the sensitivity and specificity of ovarian cancer screening. The number of such studies is limited; larger and numerous studies are necessary to further confirm the diagnostic capability.

Through fallopian tube peristalsis, protein-rich fluid from the endometrial cavity is transported into the vagina via the cervix. Protein analysis of both Pap test fluid and cervical swabs revealed similarities between these samples and tumor extracts. This establishes the first step in the feasibility of detecting ovarian cancer protein biomarkers in Pap test fluid or cervical swabs (156). Cervicovaginal fluid collected by inserting a cytobrush, similar to a Pap test into the cervix, is a useful method of detecting early changes in the fallopian tubes and their microenvironment. Rocconi et al. evaluated cervicovaginal fluid specimens by LC-MS and constructed a protein panel containing five proteins: *serine proteinase inhibitor A1*; *periplakin*; *profilin1*; *apolipoprotein A1*; and *thymosin beta4-like protein*. This panel was used as a biomarker for ovarian cancer screening to distinguish ovarian cancer patients from control groups, with an AUC of 0.86 (157).

Samples from the cervix and vagina are relatively easy to collect and acceptable to patients. Similar to cervical cancer screening, it can be integrated into routine gynecological screening. **Table 3** lists all studies that used cervical and vaginal samples for analysis. There are two main kinds of cervical cytology samples used in early detection of OC. The first is the fixative of liquid-based Pap test (145, 147, 148, 150), such as the Thinprep Cytologic Test (TCT) applied for routine screening of cervical cancer (149). The other is the brush-based Pap test (146). The obtained cervical swabs were stored in conical tubes and soaked in phosphate-buffered saline (PBS) to elute their supra-components when needed (152, 156). The prevalence of liquid-based TCT let us to hypothesize that samples extracted from the liquid-based vial are suitable for ancillary testing, ensuring both accuracy and convenience. Pap tests and cervicovaginal fluid are theoretically feasible, but their sensitivity and specificity have yet to reach expected clinical standards. In a recent study, MS and protein extension assay (PEA) were used in combination with artificial intelligence to create an 11-protein panel. This panel had a sensitivity of 97%, a specificity of 67%, and an AUC of 0.96 (158). Although this outcome did not align with the optimal differentiation achieved by the multivariate model in plasma, the researchers utilized a sample of dried, self-sampled cervicovaginal fluid (CVF) deposited on elute filter paper cards, which has been shown to provide accurate and cost-efficient screening of cervical cancer (159, 160). While the specificity of CVF compared to plasma still needs to be increased, the results offer the possibility of an ovarian cancer screening program based on self-collected CVF samples. In addition, this study also included specimens collected prior to the diagnosis of OC (before the onset of symptoms), in which the signal could also be tested, indicating that this model could provide information about future disease risk. In summary, utilizing Pap tests and cervico-vaginal fluid as liquid biopsy samples for early ovarian cancer detection offers distinct operational advantages. The diagnostic accuracy has significantly improved with advances in multi-omics technologies, demonstrating considerable clinical potential.

**Table 3:** Early detection of ovarian cancer by collecting cervical cytology or cervicovaginal fluid.

Analyte	Author, Year	No. of patients	Sample	Laboratory Technique	Biomarker /signature	Detection Rate
ctDNA	Kinde I et al., 2013 (145)	22 OC + 14 HCs	Fixative solution of liquid-based Pap smear	SafeSeqS	Detectable tumor-specific gene mutation	41% (9/22)
	Erickson BK et al., 2014 (144)	9 SA + 13 BOD	Vaginal tampon	SafeSeqS	TP53 mutation	50% (3/5)
	Wang Y et al., 2018 (148)	245 OC + 714 HCs	Liquid fixative of the Pap brush	SafeSeqS	PapSEEK	Se=33%
	Arildsen NS et al., 2019 (147)	15 HGSOC	Liquid-based archival/diagnostic Pap samples	NGS, IBSAFE	TP53 mutation	66.7% (6/9)
	Jiang X et al., 2020 (150)	19 OC	liquid-based Pap smears	WES, cSMART	cSMART multigene panel	100% (11/11)
	Krimmel-Morrison JD et al., 2020 (149)	9 III-IV HGSOC + 21 HCs	Thinprep Pap test	NGS, CRISPR-DS	tumor-derived mutation	37.5% (3/8)
	Paracchini L et al., 2020 (146)	17 II-IV; HGSOC	brush-based Pap test slides	NGS, ddPCR	TP53 somatic variants	64% (11/17)
	Paracchini L et al., 2023 (155)	117 HGSOC + 77 HCs	Pap test smears	WES	CPA (EVA test)	Se=75%; Sp=96%
	Chang CC et al., 2018 (151)	59 OC + 74 HCs	Cervical scrapings	MSP	M-index of POU4F3/MAGI2	Se=61%; Sp=62%-69%
	Wu TI et al., 2019 (152)	134 OC + 22 HCs	Cervical scrapings	qMSP	OC-risk score: methylation levels of AMPD3, NRN1, and TBX15	Se=81%; Sp=84%; AUC=0.91
Protein	Boylan KLM et al., 2021 (156)	1 HGSOC	liquid-based Pap test fixative and cervical swab	2D-LCMS	Ovarian cancer biomarkers protein	N/A
	Rocconi RP et al., 2022 (157)	33 OC + 50 HCs	cervicovaginal fluid	LC-MS	5 proteins panel	Se=64%; SP=98%; AUC=0.86
	Hedlund Lindberg J et al., 2024 (158)	116 OC + 40 HCs	Self-sampled cervicovaginal fluid	MS、PEA	11 proteins panel	Se=97%; SP=67%; AUC=0.96

SA, serous adenocarcinoma (ovarian, tubal or primary peritoneal); SafeSeqS, Safe-Sequencing System; M-index, methylation index; IBSAFE, an ultra-sensitive ddPCR method; WES, whole-exome sequencing; cSMART, circulating single-molecule amplification and resequencing technology; DS, Duplex Sequencing; 2D-LCMS, 2D-liquid chromatography mass spectrometry; CPA, copy number profile abnormality; PEA, protein extension assay.

### 2.4 Uterine and tubal lavage

Progressing in parallel with studies using Pap tests for early detection of OC is uterine cervical cells. A study detected malignant cells shed from ovarian, fallopian tubes, and peritoneal cancers by examining endometrial cytologic samples from the endometrial cavity (endometrial sampler). It is a concept similar to the detection of cervical cancer by Pap smear, but this research had a low positive detection rate of only 12% (161). Then, investigators found that using Tao brush for intrauterine sampling increased the detection rate of the diagnostic multiplex PCR-based test named PapSEEK, 45% of 51 ovarian cancer patients tested positive (148). Tumor cells shed from ovarian or endometrial cancers are carried into the uterine cavity, where the Tao brush can collect them. Consequently, the detection of exfoliated cells from high-grade-serous ovarian cancer, or precursor lesions, is a promising concept for earlier diagnosis. **Table 4** shows the studies currently used to diagnose OC by uterine lavage.

**Table 4:** Early detection of ovarian cancer by collecting samples from the uterine cavity.

Analyte	Author, Year	No. of patients	Laboratory Technique	Biomarker /signature	Detection Rate
ctDNA	Maritschnegg E et al., 2015 (162)	30 OC + 27 BGD	NGS, SafeSeqS, ddPCR	Tumor-specific mutation	80% (24/30)
	Salk JJ et al., 2019 (163)	10 HGSOC + 11 HCs	ddPCR, DS	TP53 mutations	80% (8/10)
	Ghezelayagh TS et al., 2022 (164)	20 OC +14 BGD	Ultradeep DS	TP53-specific mutation burden	N/A
	Žilovič D et al., 2023 (165)	37 HGSOC + 53 Other cases	NGS	TP53 mutations	Se=27%; Sp=100%
RNA	Hulstaert E et al., 2022 (166)	26 OC + 48 BOD	mRNA capture, small RNA sequencing	mRNA, miRNA, and exon data	Se=66%; Sp=88%
	Skryabin GO et al., 2022 (167)	5 EOC + 5HCs	Small RNA Deep Sequencing, RT-PCR	miR3753p, miR451a, miR199a-3p	Upregulated: miR3753p Downregulated: miR451a, miR199a-3p
Protein	Barnabas GD et al., 2019 (168)	49 HGOC + 127 controls	LC-MS/MS	9-protein classifier	Se=74%, Sp=66%, AUC=0.71
	Bahar-Shany K et al., 2023 (169)	24 HGOC + 164 controls (germline BRCA mutation carriers)	MS	7-protein signature	Discovery set: AUC>0.97 Validation set: AUC>0.94
Metabolites	Wang P et al., 2023 (170)	114 OC + 55 BOD	RPLC-MS, HILIC-MS	7-metabolite panel	AUC=0.957

DS, duplex sequencing; RPLC-MS, reverse-phase liquid chromatography-mass spectrometry; HILIC-MS, hydrophilic interaction liquid chromatography-mass spectrometry.

In 2015, Maritschnegg et al. firstly demonstrated that cancerous cells originating from ovarian tumors could be shed and collected through uterine lavage. Using NGS and singleplex analysis, researchers detected mutations that were consistent with those found in primary tumor tissue in 80% of OC uterine lavage samples (162). This proof-of-concept study has shown the potentially diagnostic power of the uterine lavage method for OC detection, especially for early detection in high-risk populations. Analysis of uterine lavage samples using NGS reveals TP53 mutations in about 60% of OC patients (162, 165). Since previous studies have been performed based on known mutated genes in the primary tumor lesion, Salk et al. combined Uterine and tubal lavage (Utl) with Duplex Sequencing (DS), which has a higher sensitivity (80%) for detecting true-positive cancer-derived TP53 mutations in HGSOC without prior knowledge of the tumor mutation (163).

Current transcriptomic studies utilizing anatomically proximate fluids remain theoretical. A proof-of-principle extracellular transcriptomic analysis utilizing messenger RNA capture and small RNA sequencing revealed that the lavage fluid had ovarian and fallopian tube-specific mRNA enrichment (166). This study first applied RNA-seq to utero-tubal lavage samples, yielding a multi-omics classifier based on combined mRNA, miRNA and exon data. With 66% sensitivity and 88% specificity, the model demonstrates technical feasibility for RNA isolation and sequencing from utero-tubal lavage fluid, revealing ovarian and fallopian tube-specific mRNA signatures useful for early diagnosis. Skryabin et al. also confirmed the differences in the expression levels of miRNAs between healthy individuals and EOC patients are particularly associated with cancer, such as miR-200 family members (167). Despite the lack of reliable cohort study data demonstrating the value of its application, RNA in utero-tubal lavage fluid is 8 times higher than in platelet-free plasma (166) and remains a potential biomarker that can be used for the early detection.

Also, the protein and metabolites in the uterine fluid have the potential to provide a broader range of biomarkers for early detection. Combining deep microvesicle proteomics with gynecologic intracanal fluid biopsy, support vector machine algorithms were applied to generate a 9-protein classifier with 70% sensitivity and 76% specificity. The signature correctly identified all Stage I lesions (168). As the expression profile of BRCA-mutated Müllerian epithelium is significantly different from the WT pattern, the team further characterized the proteomic signatures to identify HGSOC in BRCA carriers. The 7-protein panel discriminated between patients with high-risk germline BRCA mutations and controls with an AUC >0.97 and a negative predictive value of 100%. In addition, the questionnaire results reported that this sampling method is clinically acceptable with favorable pain scores and safety (169). As for metabolomics, Wang et al. revealed the metabolomic profile of uterine fluid and developed a panel of 7 metabolites that can discriminate women with benign gynecological diseases from those with early-stage OC with obscure symptoms. The AUC of this panel was 0.957, significantly higher than 0.817 for CA125 and 0.841 for ROMA, providing an accurate and sensitive strategy for the early diagnosis of OC (170).

Depending on the different types of catheters, current UtL collection approaches can be categorized into three types: one-way, two-way, and three-way catheters. One-way catheter, such as intrauterine insemination catheter and rigid pipelle uterine samplers (166, 168, 169), was inserted transcervically into the uterine cavity. The saline was flushed directly and retrieved immediately. The two-way catheter is primarily composed of existing catheters, such as dual-channel catheters (167), two-way hysterosalpingography catheter (165), and size ten (10 F) Foley catheter (170), that are primarily used for other purposes. The balloon was inflated with saline to seal the cervical canal and prevent retrograde leakage of saline. The normal saline was slowly infused into the uterine cavity through the catheter tube, and left for a while before gently suctioning to collect the liquid. The uterine cavity is very small and the anterior and posterior walls lie on top of each other. When using a single-channel catheter, it was removed immediately after saline injection, the saline could not adequately and completely irrigate the uterine cavity. Besides, the injected saline tended to flow back into the vagina. The use of a two-channel catheter with a balloon solved the problem of saline reflux, but did not ensure that the sample collected could be fully representative of the internal environment in the uterine cavity. In order to solve the above problems, the novel three-way catheter was designed and developed (171). There are two lavage channels, each with two openings, one on the tip of the catheter facing forward and one at the side. The third tube is the balloon channel carrying a valve. Two syringes, one of them containing saline, are connected to the two lavage tubes. By pushing on the plunger of the syringe containing saline, the uterine cavity and fallopian tubes were slowly perfused. Simultaneously, the plunger of the empty syringe was gently pulled out. The clinical utility of the three-way catheter was evaluated on whether lavage solution could be successfully obtained, how easy it was to insert, whether cervical dilation was required, the volume of lavage fluid collected, and the amount of DNA extracted. Moreover, the study assessed the pain level, time required for placement, and other complications in patients compared to intrauterine device (IUD) placement. It has been proven that this three-way catheter caused minimal pain, both in terms of intensity and duration, making it a practical and safe option.

Currently, using uterine lavage fluid as a liquid biopsy sample, the sensitivity of OC early detection ranges from 70% to 80%. The detection accuracy for tumor-associated TP53 mutations in uterine lavage reached approximately 80%, higher than the Pap test. These findings support the clinical value of proximal liquid biopsy for improving detection rates. Recent research has established that most HGSOCs originate from epithelial precursor lesions on the fallopian tubes rather than ovarian tissue (172). Serous tubal intraepithelial carcinoma (STIC) is now recognized as the direct precancerous lesion preceding HGSOC development. Mutational evolutionary analyses identify a 6-year interval between the TP53-mutated precursor emergence and the initiation of HGSOC (172). Tumor cells and related substances from precancerous or early OC lesions can transit through the tubal ducts into the uterine cavity. These can be detected directly in utero-tubal lavage fluid, where they are more abundant than circulating tumor cells in the blood, facilitating early OC detection and diagnosis. In summary, there are limited studies on the detection of ovarian cancer using uterine lavage, which predominantly employ sequencing to analyze tumor-associated genes, such as TP53, yet the detection accuracy remains unsatisfactory. The application of multiple MS-based assays of proteins or metabolites in uterine lavage fluid, in conjunction with machine learning algorithms, has markedly enhanced the diagnostic efficacy. This may prove to be a fruitful avenue for future research and development.

## 3 Discussion

With the accelerating development of multi-omics technologies, especially in combination with the application of machine learning algorithms, early detection of cancer has evolved from a single level of tumor-associated gene mutations to an integrated multi-omics analysis. More and more studies have supported the potential of liquid biopsy in the discovery of candidate biomarkers. This has facilitated the progression of the early detection of OC. There are commercially available liquid biopsy-based platforms for early detection of ovarian cancer, such as CancerSEEK, PapSEEK, OvaPrint^TM^, and MCED. CancerSEEK is a liquid biopsy platform designed for early detection of eight cancer types. It demonstrates 98% sensitivity for ovarian cancer (25). PapSEEK is a diagnostic multiplex PCR-based test. It utilizes Pap brush and Tao brush samples to detect 18 mutations highly associated with endometrial and ovarian cancers for early diagnosis (148). OvaPrint^TM^ is a cfDNA mehylation liquid biopsy platform to discriminate benign pelvic masses from HGSOC preoperatively (14). Multicancer early detection (MCED) blood tests can detect cancer signals from cfDNA through detection of cancer-specific DNA methylation. PATHFINDER, a prospective cohort study, enrolled 6,662 participants aged 50 years or older without signs or symptoms of cancer in MCED testing (173). In cases with positive MCED results and confirmed cancer diagnoses, the testing accurately predicted tumor origin and significantly reduced time to diagnostic confirmation, demonstrating the clinical feasibility of this approach. Among the 35 true-positive participants, there was only one case of ovarian cancer, which was stage III. CancerSEEK and MCED are platforms for pan-cancer early detection. The updated results from CancerSEEK are still pending. Researchers are currently conducting larger-scale trials based on the PATHFINDER study to evaluate MCED testing. Regarding PapSEEK and OvaPrint™, validation studies for ovarian cancer early detection in wider populations are still lacking. Thus, whether these platforms are applicable for early detection of OC remains unknown.

It is not easy to make side-by-side comparisons between samples, as differences in the baseline of the different samples, the use of the detection assays, and the discovery of different potential markers will inevitably lead to differences in the accuracy of the assays. **Table 5** summarizes the collection methods, respective advantages, current challenges, and target populations of different liquid biopsy samples for early detection of ovarian cancer. The use of plasma/serum for early detection of ovarian cancer leads to relatively satisfactory performance. Peripheral blood represents a convenient and easily manageable biospecimen with high patient compliance, making it particularly suitable for long-term monitoring in general populations. However, it remains debatable whether blood-based assays can truly detect OC at an early stage and reduce mortality. Technical challenges in analyzing low-concentration circulating tumor components drive the need for sophisticated detection platforms, with consequent economic implications for diagnostic implementation. As for urine, the clinical manifestations of relevant potential biomarkers are not sufficient for the effective detection of early OC, and existing studies cannot prove their authenticity and reliability for OC early screening. Interestingly, the fluorescent sites in urine are unique, and monitoring urine autofluorescence may offer new opportunities for the development of ovarian cancer screening methods (127).

**Table 5:** Comparative analysis of liquid biopsy specimens for ovarian cancer early detection.

Sample	Collection	Advantages	Challenges	Clinical application
Serum/Plasma	2–10 ml of peripheral blood	Most extensively studied Convenient Favorable for clinical follow-up High patient acceptance Superior diagnostic accuracy	Low abundance in early-stage disease Requires sophisticated high-throughput techniques	Screening and early detection tool for the general population
Urine	Morning urine sample	Convenient and rapid Non-invasive Can be collected in quantities More stable Urine autofluorescence serves as a unique biomarker	Limited relevant research to date Insufficient diagnostic performance Absence of clinical cohort verification	Insufficient evidence for clinical application
Pap test and cervicovaginal fluid	The fixative of the liquid-based Pap test Cervical swabs stored in tubes	Methods of routine screening for cervical cancer Anatomically adjacent Detect more tumor components than the peripheral blood Relatively convenient to collect and acceptable Self-sampled CVFs are more convenient	Inadequate sensitivity and specificity for clinical applications Requires sophisticated techniques	Potential for screening and early detection in the general population
Uterine and tubal lavage	one-way, two-way, and three-way catheter to collect 5–10 ml of lavage	Anatomically closer to the tumor origin Provides more comprehensive diagnostic information than the Pap test and cervicovaginal fluid	Intrusive operation Lack of standardized tools	Early detection and monitoring tool for high-risk populations

**Figure 2:**
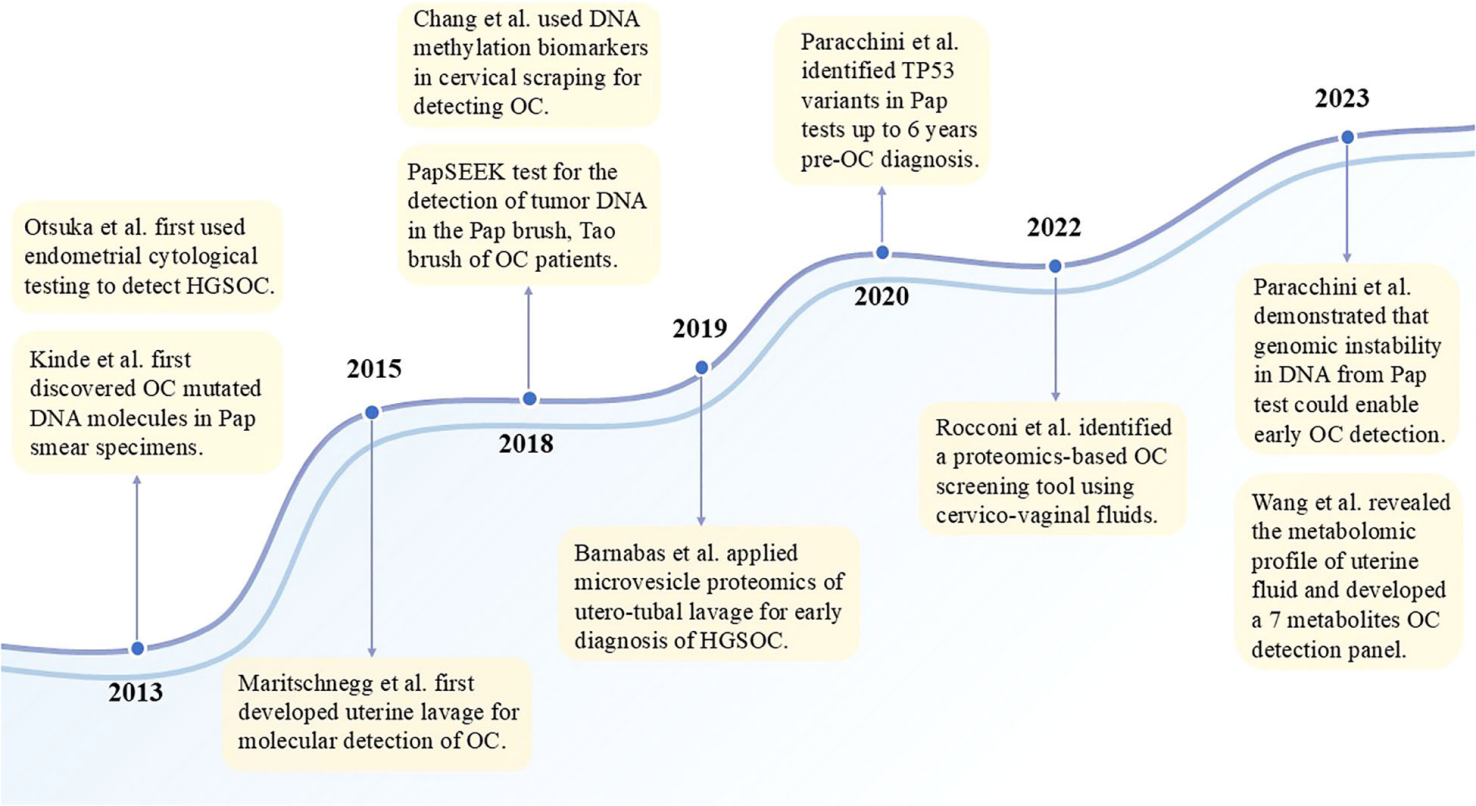
Chronological development of proximal liquid biopsy in ovarian cancer research. Since 2013, when Pap tests and uterine lavage were first shown to detect ovarian cancer, multi-omics studies of proximal liquid biopsies have aimed to develop practical early detection tools with high sensitivity.

Proximal liquid biopsy is an assay that takes the sample directly from the body cavity where the tumor is located, increasing the likelihood of detecting early or even precancerous lesions. The entire female genital tract is a connected lumen. Collection of biospecimens from cervicovaginal fluid and uterine lavage is clinically termed proximal fluid biopsy. Using cervical smears to diagnose OC is a relatively new approach that has emerged in the last decade, similar to using cervicovaginal fluid. **Figure 2** illustrates a chronological timeline from 2013 to the present, demonstrating landmark studies of proximal liquid biopsy in ovarian cancer detection. To meet the high sensitivity and specificity required for early diagnosis of OC, the performance of such specimens is currently inadequate. It is encouraging to note that the latest studies use more accurate high-throughput technology combined with artificial intelligence to develop tests that could significantly improve the diagnostic performance of cervical cells and cervicovaginal fluid. For example, Hedlund Lindberg J et al. achieved a sensitivity of 97% using self-collected cervicovaginal fluid on paper cards as the sample. Although the specificity is not the most satisfactory, it offers the possibility of using self-collected samples for ovarian cancer screening programs (158). With further technological advancements, we believe these specimens have promising potential to be used for OC early diagnosis in the future, similar to cervical cancer screening. The uterine cavity is anatomically closer to where the tumor originates, and components obtained from it could theoretically provide more complete and abundant diagnostic information, further increasing the likelihood of detecting early-stage or even precancerous lesions. Practically, uterine lavage has also demonstrated superiority and accuracy over cervical/vaginal cytology. This assay has a high diagnostic potential, but is not a completely non-invasive sampling method. It is uncomfortable for patients and carries a theoretical risk of infection to some extent, making it unsuitable for universal screening of healthy populations. For carriers of germline BRCA mutations, uterine lavage could be a viable monitoring option. The general population of women should be divided into the population with high risk and the population with an average risk of ovarian cancer. Because of the absence of screening tools with excellent sensitivity and specificity at present, bilateral salpingo-oophorectomy remains the standard risk-reduction strategy for high-risk women, particularly BRCA1/2 mutation carriers, typically recommended between the ages of 35–45 after completing their reproductive plans. Although risk-reduction bilateral salpingo-oophorectomy (RRSO) has been confirmed to significantly decrease the risk of BRCA1/2-associated ovarian or fallopian tube cancer, and consequently reduce mortality, many high-risk individuals have declined or postponed the procedure due to menopause and the subsequent health consequences of early surgery. For this population, uterine lavage, combined with a superior diagnostic performance biomarker panel, can be administered semiannually as a tool for long-term surveillance when a deferred RRSO is requested or required (169).

However, a gap persists between discovery research and practical clinical application, with several challenges need to be addressed. First, ovarian cancer is highly heterogeneous. Because of the predominance of EOC, current studies either focus only on EOC, even HGSOC, or the study cohorts have a small percentage of other OC subtypes, which are not well represented. OC is typically asymptomatic in its early stages, with most patients presenting at advanced stages when they come for the initial consultation. Consequently, advanced-stage OC accounted for a large proportion of the cohorts, and the majority of early-stage OC are type I or borderline tumors. Importantly, the paucity of stage I and STIC lesions hinders the evaluation of liquid biopsy techniques and classifiers for genuinely detecting clinically latent OC. Also, there is a lack of multicenter prospective studies in large, multi-ethnic populations. Most of the research has only evaluated the diagnostic performance of candidate biomarkers/biomarker panels, and a small proportion of which has examined the relationship between candidate panels and age. Further work is needed to explore the correlations and interactions of candidate biomarkers with other ovarian cancer risk factors, such as prior chemotherapy, endometriosis, or germline BRCA mutations. Lastly, both the collection of uterine lavage fluid and the extraction of tumor-associated fractions in the laboratory do not have standardized workflows, which severely limits the reproducibility of assays. Future efforts should develop optimal standardized procedures and analysis platforms to validate new technologies and prospective biomarkers in robustness and reproducibility.

In conclusion, liquid biopsy has emerged as a promising option for screening and early detection of OC. The minimally invasive and rapid nature meets the requirements for screening in healthy populations. Various body fluid specimens have their strengths and weaknesses; blood, cervical cytology, cervicovaginal fluid, and uterine lavage could be potential specimen sources for screening and early diagnosis of OC in different risk groups. Hopefully, the selection of appropriate liquid biopsy samples, the application of multi-omics technology for analysis, and the combination of artificial intelligence and machine learning algorithms will improve OC early detection and contribute to management.
